# Do elections accelerate the COVID-19 pandemic? Evidence from a natural experiment

**DOI:** 10.1007/s00148-021-00870-1

**Published:** 2021-09-17

**Authors:** Ján Palguta, René Levínský, Samuel Škoda

**Affiliations:** 1 Carlos III University of Madrid (UC3M), Department of Economics, c/ Madrid 126, 28903, Getafe, Madrid Spain; 2 Center for Economic Research and Graduate Education – Economics Institute (CERGE-EI), A joint workplace of Charles University in Prague and the Economics Institute of the Czech Academy of Sciences, Politických vězňů 7, 111 21 Prague, Czech Republic; 3 Centre for Modelling of Biological and Social Processes (BISOP), Na Břehu, 497/15, 190 00 Prague, Czech Republic; 4 University of Zurich, Department of Economics, Schönberggasse 1, 8001 Zurich, Switzerland

**Keywords:** Election, COVID-19, Natural experiment, Event study, D70, D72, H0, H12, H75, I10, I18

## Abstract

Elections define representative democracies but also produce spikes in physical mobility if voters need to travel to polling places. In this paper, we examine whether large-scale, in-person elections propagate the spread of COVID-19. We exploit a natural experiment from the Czech Republic, which biannually renews mandates in one-third of Senate constituencies that rotate according to the 1995 election law. We show that in the second and third weeks after the 2020 elections (held on October 9–10), new COVID-19 infections grew significantly faster in voting compared to non-voting constituencies. A temporarily related peak in hospital admissions and essentially no changes in test positivity rates suggest that the acceleration was not merely due to increased testing. The acceleration did not occur in the population above 65, consistently with strategic risk-avoidance by older voters. Our results have implications for postal voting reforms or postponing of large-scale, in-person (electoral) events during viral outbreaks.

## Introduction

The dramatic onset of the COVID-19 pandemic, just like other large-scale emergencies, disrupted lives, businesses and communities worldwide as governments responded with extraordinary measures to battle the spread of the virus. While non-pharmaceutical interventions that limit mobility and isolate potentially infected people are designed to keep the pandemic at bay, they also clash with the cornerstone of democracy: regular, free and fair elections.

The public discourse over holding massive, in-person elections amid the growing pandemic has been highly contentious in numerous countries. Early academic literature promptly attempted to quantify the impact of elections on the growth in new infections and mortality (Berry et al. 2020; Feltham et al. 2020; Leung et al. 2020), but, contrary to widespread fears, found little evidence that elections would speed up pandemic growth. Concerns about these estimates included questions related to choosing the correct model of counterfactual pandemic evolution and the lack of exogenous variation in voter participation in elections. At the same time, evidence by Bertoli et al. (2020), Cotti et al. (2021), and Cassan and Sangnier (2020) hinted, in a stark contrast, that elections can be associated with significant speeding up of the pandemic. These conflicting results make the challenge of defining a credible counterfactual in which one could track the same area (e.g. a country) simultaneously holding and abstaining from a massive electoral event even more salient.^1^

In this paper, we provide evidence from a natural experiment that allows us to convincingly estimate the causal impact of large-scale, in-person elections on the COVID-19 pandemic without relying on strong assumptions about voter turnout and pre-electoral pandemic trajectories. Our study is based on constituency-level variation from Czechia, a developed, high-income, EU-member country that biannually renews mandates in one-third of Senate constituencies, which rotate deterministically in holding elections according to the country’s 1995 election law. The constituencies voting in each turn are geographically scattered across the country. The first round of Senate elections is always organized jointly with municipal or regional elections, which are held nationally. The second round, however, held 1 week after the first round, is not combined with another nationwide electoral event. This institutional setting implies that in the absence of the second round of the 2020 elections (held on October 9–10), the pandemic would evolve along parallel paths in voting and non-voting constituencies because the initial pre-electoral pandemic conditions in constituencies should be uncorrelated with their assignment to the 2020 electoral turn.

In our results, we show that in the second and third weeks after the elections, the growth rates in new COVID-19 infections are significantly higher in voting compared with non-voting constituencies. For illustration, the 14-day growth in new cases is 24.6 percentage points higher in the third week after elections in constituencies that just held elections compared with the baseline 146.4% growth rate over the last 14 days in non-voting constituencies. A temporarily linked acceleration in hospital admissions (significant at the 5% level at its peak in the third week) together with essentially no changes in test positivity rates indicate that the acceleration does not merely reflect increased testing in voting constituencies.

We validate our findings using standard event study tests of no differences in pre-trends across voting and non-voting constituencies. We also implement a battery of tests showing no initial differences in COVID-19 prevalence, active cases, reproductive number R and a number of socio-demographic outcomes characterizing economic status, education and age structure of the population across constituencies. Using data from earlier regional elections, we find no differences across constituencies in terms of voter electoral preferences and turnout. Our event study estimates are robust to controlling for the earlier epidemiological situation in constituencies and any observed and unobserved time-invariant factors.

In our heterogeneity analysis, we find that the pandemic acceleration is only present in the population younger than 65 and is absent in older cohorts, consistent with strategic risk-avoidance by older voters (Dave et al. 2020b), who are at greater risk of hospitalization or dying if diagnosed with COVID-19 (Williamson et al. 2020). Also, we find that the acceleration is significantly higher in municipalities with a below-median share of individuals with at least secondary education. The evidence supports the literature suggesting that socio-economic factors play an important role for the speed of the pandemic’s spread and its mitigation (Wright et al. 2020).

To inspect the mechanism of viral spread, we use data from Google and Apple and estimate increased mobility and shorter stays at places of residence during elections. In comparison, we do not find significant spikes in mobility at other types of locations, namely, in retail and recreation facilities, groceries and pharmacies, parks, transit stations and workplaces. Using data from a unique representative panel survey, we also do not find respondents to be more likely to engage in more frequent family reunions, restaurant visits, group holidays or large public events (including electoral rallies) in the election week. We only estimate the respondents were more likely to commute by crowded public transport, likely because they needed to get to polling places.

Our analysis contributes to several strands of the literature. First, we add to the rich and growing literature that evaluates public health measures aimed at reducing the spread of infectious viral diseases. Earlier studies in this strand find that policies aimed at reducing population flows by restricting mobility (Fang et al. 2020; Qiu et al. 2020), adopting safer-at-home orders and non-essential business closures (Amuedo-Dorantes et al. 2021), school closures (Adda 2016; Litvinova et al. 2019) or paid sick leaves that keep contagious workers at home (Barmby and Larguem 2009; Pichler and Ziebarth 2017) can mitigate viral transmission. Along this line, holding large gatherings of attendees with little or no social distancing has been also suspected to contribute to viral spread, especially to the COVID-19 pandemic (McCloskey et al. 2020). Earlier studies examine the epidemic impact of a large motorcycle event (Dave et al. 2021), the role of New York City subways (Harris 2020), the impact of college students returning after spring break (Mangrum and Niekamp 2021) and the role of Black Lives Matter protests (Dave et al. 2020b) .

We are aware of several studies that focus explicitly on elections but deliver conflicting results. In particular, Leung et al. (2020), Berry et al. (2020), and Feltham et al. (2020) find no effects of the 2020 US primary elections on COVID-19 transmission and mortality. In a stark contrast, Cotti et al. (2021) find in the same setting of primary elections in Wisconsin that a higher number of in-person voters per polling station is linked with higher positivity 2–3 weeks later. In the European context, some studies of municipal elections in France suggest that higher voter turnout is associated with higher post-electoral hospitalizations (Cassan and Sangnier 2020) and mortality (Bertoli et al. 2020), whereas other studies find no impact (Duchemin et al. 2020; Zeitoun et al. 2020).

The common features of these studies are the challenges associated with choosing the correct model of the counterfactual pandemic evolution and the difficulty of addressing non-random voter turnout. In this respect, Cotti et al. (2021) abstain from causal language as they recognize that the number of polling stations per voter might be endogenously set. Bertoli et al. (2020) predict turnout using the intensity of local electoral competition, but, as pointed out by Bach et al. (2021), their estimates are attributable to measurement error. Other studies either assume turnout to be fully exogenous with respect to pre-electoral pandemic conditions or do not discuss that many socio-economic determinants of turnout might also shape the trajectories of pandemic spread and compliance with mitigation policies.^2^ Unlike previous work, our study provides evidence from a clear natural experiment that does not require complex modelling of pre-electoral pandemic trajectories nor strong assumptions about voter turnout to convincingly estimate the causal effect of massive (electoral) event on viral spread.

Next, our analysis adds to the growing literature on socio-economic determinants of viral propagation. In this strand, Adda (2016) finds pro-cyclical effects of economic activity and inter-regional trade on viral transmission. Markowitz et al. (2019) show that higher employment is linked with higher incidence of influenza. Using data from the early stages of COVID-19 pandemic in China, Qiu et al. (2020) show that cities with higher GDP per capita had higher transmission rates, which is ascribed to more social interactions as economic activities increase. As pointed out by Dave et al. (2020b) and Gupta et al. (2020), however, individuals can strategically respond to the perceived risk of contagion, which can slow down viral propagation. In line with this argument, Wright et al. (2020) show that residents of high-income areas in the US comply with shelter-in-place ordinances much more than their counterparts in areas with lower incomes, even after accounting for partisanship, population density and unemployment. We contribute to this literature by showing that pandemic acceleration due to elections was significantly higher in municipalities with a below-median share of individuals with at least secondary education and absent in the population above 65, which supports the strategic risk-avoidance hypothesis.

Finally, we contribute to the studies on the electoral implications of viral outbreaks. In this strand, Pulejo and Querubín (2021) find that incumbents who can run for reelection implement less stringent anti-pandemic restrictions when elections are closer in time. Amuedo-Dorantes et al. (2021) moreover suggest that political ideology might compromise the efficacy of non-pharmaceutical interventions (NPIs) as the adoption speed of NPIs during the COVID-19 crisis appeared to be less effective in Republican counties. In line with the broad literature on retrospective voting (reviewed by Ashworth 2012), Warshaw et al. (2020) show that US states and local areas with higher COVID-19 fatality rates were less likely to support President Trump and Republican candidates for the House and Senate. Baccini et al. (2021) argue that the prevalence of COVID-19 likely decided the 2020 US presidential election. We add to this literature by arguing that in the case of strategic risk-avoidance by vulnerable groups of voters, pandemic outbreaks can produce a side effect of elevated absenteeism of important socio-economic groups in elections. In our setting, we estimate little pandemic acceleration due to elections especially in the population above 65. We also estimate that the share of population above 65 is less strongly associated with voter turnout in 2020 compared with the previous 2016 elections. Our evidence is therefore suggestive of self-imposed restrictions on electoral franchise by older voters, which has important policy implications for the organization of elections during viral outbreaks.

The rest of the paper is structured as follows. Section 2 describes the institutional background and data. Section 3 outlines the empirical methodology. Section 4 presents our findings. Section 5 summarizes, discusses policy implications and concludes.

## Background and data

### Institutional setting

The Czech Republic is a developed, high-income, Central European country, which has been a member of the European Union since 2004 and OECD since 1995. The Czech parliament is bicameral, consisting of the Chamber of Deputies and of the Senate with 81 members elected in single-seat constituencies. The role of the Senate is relatively limited, primarily restricted to vetoing bills approved by the Chamber of Deputies and confirming judges of the Constitutional Court. Its vetoes can be overruled unless the matter relates to constitutional law, electoral law or an international treaty.

**Fig. 1:**
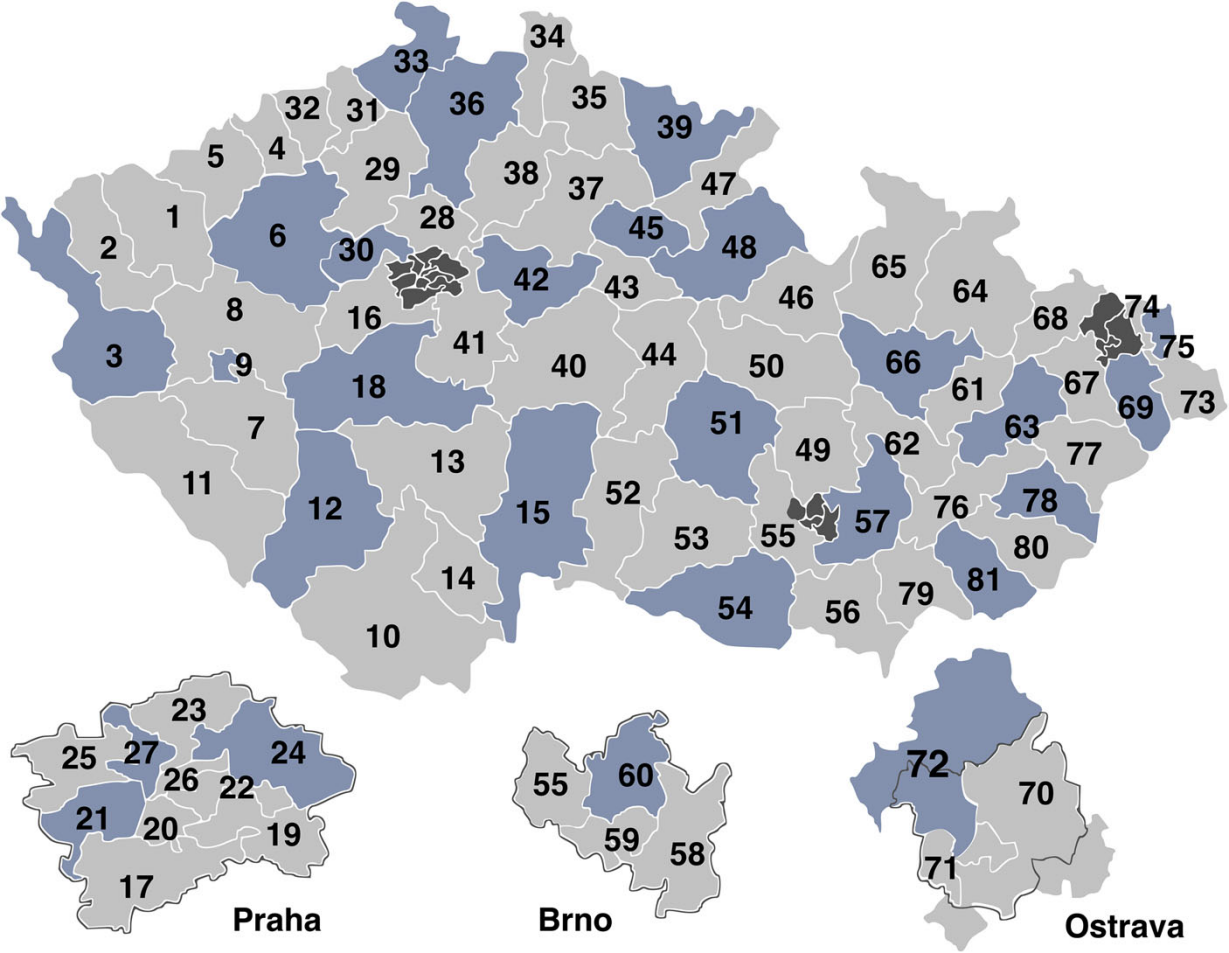
Senate constituencies in the Czech Republic. Only constituencies with pre-assigned numbers 3, 6, 9, 12, ... (in blue) voted in the 2020 Senate election. Constituencies within the three largest Czech cities are shown separately

The members of the Senate serve for 6 years, with one-third of the Senate being reelected every 2 years, in a similar fashion to the US Senate. The constituencies that are up for reelection are predetermined by the election law of 1995, which assigns every constituency a fixed number, from 1 to 81, roughly from the western to the eastern part of the country (see Fig. 1). The division into constituencies is permanent and does not correspond to a specific level of local government.

The law perfectly determines constituencies in which Senate elections take place in a given election year. Specifically, these are stable, regular rotations of constituencies enumerated $1-4-7-\dots -79$, $2-5-8-\dots -80$ and $3-6-9-\dots -81$. As it is evident in Fig. 1, this rule effectively guarantees that constituencies in each of the three rotations are geographically scattered across the country, in no particular pattern. Therefore, while every 2 years only one-third of constituencies votes, the law defines constituencies in such a way that the whole country is represented in each turn.

The Senate elections consist of two-round runoff voting. The first round is always organized jointly with either municipal or regional elections, which are held nationally.^3^ The second round of the Senate elections, held exactly 1 week after the first round, is not combined with another nationwide electoral event, making it an ideal natural experiment for our question. Hence, our focus is on the second round of the 2020 Senate election held on Friday and Saturday of October 9–10.

In all Czech elections, regardless of the elected office, voters need to vote in person. For instance, voters located abroad at the time of elections need to visit Czech embassies if they wish to cast votes. In the 2020 turn, unprecedentedly impacted by COVID-19 pandemic, voters in quarantine and mandatory isolation could participate in elections by voting in one of 78 (resp. 44) drive-in stations set-up for the first (resp. second) round (IFES 2020). However, this option was used by very few voters (3,672 and 422 voters in the first and second round, respectively). Voters unable to attend to the drive-in sites could also request mobile ballot boxes to be delivered to their place of residence, but such requests needed to be made at least a day before elections.

As a result of the first round, the second round of Senate elections was held in 26 out of 27 constituencies from the 2020 turn. One constituency in Děčín had a winner declared already in the first round. The turnout in the second round was 16.7% compared to the 38.0% in the first round. This is not an irregular drop in turnout from the perspective of the previous Senate elections. For example, since 2012 the turnout in the first round of the Senate elections has always ranged between 33.5 and 42.3%, while only 15.4–18.6% of voters attended to the second round. The turnout was relatively even across constituencies, ranging between 10.8 and 25.5%, with the exception of Karviná, where the turnout was 7.7% (CZSO 2020).

### Pandemic situation

The first wave of the COVID-19 pandemic in Europe that started in March 2020 was relatively mild in the Czech Republic. In a stark contrast, the Czech Republic has been among countries most affected by the second autumn wave of the pandemic, becoming the leading EU country in terms of new infections per capita, approximately at the time the second round of Senate elections (Roser et al. 2020).

In Fig. 6 in the Appendix we plot the growth of daily new cases since August 2020, with each round of the Senate elections denoted by a vertical line. In Fig. 7 in the Appendix we plot total active hospitalizations and cumulative deaths. Table 6 in the Appendix summarizes the positions of all parties represented in the Czech Parliament on the issue of anti-pandemic interventions. Finally, in Table 7 in the Appendix we present the timeline of anti-pandemic measures adopted by the government. In sum, we can observe that despite of the 14-day incidence approximately doubling every 10 days in early September, the government was reluctant to adopt strict interventions that might have slowed down the pandemic. All parliamentary parties in favour of strict interventions were either in opposition or had little bargaining power to push through their proposals.

At the time of elections, the most relevant anti-pandemic measures thus included restrictions on attendance above 50 people in public events without assigned seats, mandatory earlier closing hours in restaurants and mandatory use of face masks in public transport and indoors (with the exception of private housing and classrooms). Large-scale electoral rallies thus were not allowed in the last weeks before elections. On the election day, masks were mandatory at polling places, but needed to be temporarily put down to enable identification. Voters were instructed to use hand disinfectants and follow social distancing protocols. Voters were also encouraged to bring their own pens, although markers were provided also by electoral committees (IFES 2020).

### Data

In our analysis, we combine epidemiological, electoral, socio-demographic and physical mobility data to study the relationship between organizing large-scale, in-person elections and propagation of the COVID-19 pandemic.^4^

#### Epidemiological data

The epidemiological data from the Czech Ministry of Health and the Institute of Health Information and Statistics describe daily pandemic situation in all 6,259 Czech municipalities, starting from the first COVID-19 cases in March 2020. The data contain information about active cases, daily incidence of new cases and cases specifically in the population above 65, enabling us to calculate total cumulative cases (prevalence) and pandemic growth rates at any date over any arbitrarily long period for the whole population as well as the population above 65. We specifically calculate the growth rates over 7, 14 or 28 days, which enables us to avoid weekly cycles in COVID-19 incidence. The 7- and 14-day incidence rates are also among the most typical measures for communicating the COVID-19 development to the public. The data on new cases are attributed to municipalities based on the place of permanent residence.

The epidemiological data further include daily information about active COVID-19 hospitalizations and PCR test positivity rates in 206 communes (*obce s rozšířenou působností*), an administration unit which is one level above that of a municipality. We use the hospitalization data to examine whether new infections translate into health outcomes that are unlikely to be dependent on country-specific approach to detecting and reporting COVID-19 cases. PCR positivity data enable us to see to what extent the growth in new cases is associated with changes in the intensity of testing.^5^

#### Electoral data

We merge the epidemiological data with electoral data from the Czech Statistical Office which classify municipalities into Senate constituencies.^6^ When working with commune-level data, we calculate the share of population that belongs to constituencies voting in the 2020 Senate elections. The reason is that municipalities belonging to the same commune may belong to different constituencies, including those where Senate elections were not held in 2020.

The electoral data includes also results from 2020 regional elections, which were held together with the first round of the Senate elections, yielding additional information about voter preferences just 1 week prior to our natural experiment. We use this additional data only to check balance in voter preferences and earlier turnout across constituencies prior to the second round of the Senate elections.

#### Socio-demographic data

We complement our data with socio-demographic variables characterizing the economic status (employed, unemployed, out of labour force), education level and the age structure of the Czech population. The data are from the 2011 Population and housing census implemented by the Czech Statistic Office. The data was collected at the individual level, but is provided publicly as averages in municipalities. We use this data in our heterogeneity analysis and as a way to check balance in average municipal characteristics across voting and non-voting constituencies.

#### Mobility data

Finally, we use daily mobility data from Google and Apple, which respectively describe physical mobility according to 6 different categories of locations (retail and recreation, grocery and pharmacy, parks, transit stations, workplaces, residential areas) and 3 modes of transportation (walking, driving, transit). In addition, we use finer weekly data from a unique representative panel survey “Life during the pandemic” which examines social activities of $\sim $2,200 Czech households since mid-March 2020. The survey includes questions about the frequency of the use of crowded public transport, family visits, restaurant visits, group holidays, and attendance at large public events. Table 9 in the Appendix gives exact wording of the examined survey questions.

#### Summary statistics

Table 1 provides summary statistics. The means and standard deviations are reported for all municipalities as well as for municipalities in voting and non-voting constituencies separately. The reported *t*-tests suggest little differences across voting and non-voting constituencies in terms of the pre-electoral pandemic situation, voter preferences towards political parties, and voter turnout in the earlier regional elections. At the same time, the municipalities are strikingly similar in terms of the economic status, education and the age structure of the population.^7^ The lack of significant differences across voting and non-voting constituencies strongly supports the validity of our identification strategy.

**Table 1:** Summary statistics

	All constituencies			Voting in 2020			Non-voting in 2020			
	*N*	Mean	Std.Dev.	*N*	Mean	Std.Dev.	*N*	Mean	Std.Dev.	*t*-test
Pandemic situation during the second round of Senate elections (on October 9, 2020)										
All cumulative cases (prevalence) per 100k	6,259	782.25	889.11	2,134	743.70	811.24	4,125	802.20	926.29	0.348
Active cases per 100k	6,259	336.76	506.55	2,134	323.81	513.14	4,125	343.46	503.03	0.555
Hospitalized per 100k	206	19.77	14.66	.	.	.	.	.	.	.
Reproductive number *R*	2,621	1.66	1.65	870	1.62	1.65	1,751	1.68	1.65	0.477
Inspected outcomes (on October 9, 2020)										
(Prev (t) - Prev (t-14)) *100 / Prev (t-14)	6,259	71.77	149.43	2,134	69.05	142.37	4,125	73.18	152.96	0.592
(Prev (t) - Prev (t-7)) *100 / Prev (t-7)	6,259	40.30	82.62	2,134	37.99	81.40	4,125	41.50	83.22	0.371
(Hospit (t) - Hospit (t-14)) *100 / Hospit (t-14)	206	161.45	240.08	.	.	.	.	.	.	.
(Hospit (t) - Hospit (t-7)) *100 / Hospit (t-7)	206	86.86	163.32	.	.	.	.	.	.	.
Regional election outcomes (on October 2–3, 2020)										
Turnout	6,251	40.92	8.99	2,129	40.32	8.59	4,122	41.23	9.18	0.325
Electoral vote shares (%):										
ANO 2011	6,251	21.86	8.42	2,129	21.28	8.14	4,122	22.16	8.55	0.434
Civic Democratic Party (ODS)	6,251	13.16	8.26	2,129	8.26	12.34	4,122	13.59	8.34	0.331
Czech Pirate Party	6,251	12.60	5.53	2,129	12.95	5.87	4,122	12.42	5.34	0.420
Czech Social Democratic Party (ČSSD)	6,251	7.53	6.96	2,129	7.83	6.99	4,122	7.37	6.94	0.715
Freedom and Direct Democracy (SPD)	6,251	5.85	3.86	2,129	5.86	3.88	4,122	5.84	3.85	0.972
Communist Party (KSČM)	6,251	5.36	4.16	2,129	5.44	4.01	4,122	5.32	4.23	0.753
Economic status (%)										
Employed	6,198	39.55	4.64	2,118	39.55	4.51	4,080	39.55	4.71	0.998
Unemployed	6,156	4.94	2.13	2,097	4.98	2.00	4,059	4.92	2.20	0.843
Out of labour force, elderly, children	6,089	55.54	4.15	2,078	55.52	4.24	4,011	55.55	4.11	0.909
Education category (%)										
Younger than 15	6,042	15.05	3.04	2,058	15.05	2.98	3,984	15.05	3.07	0.973
Completed elementary school	6,042	18.46	4.90	2,058	18.81	4.93	3,984	18.27	4.87	0.371
Completed high school	6,042	56.81	4.80	2,058	56.55	4.64	3,984	56.94	4.88	0.385
Completed college	6,042	6.28	3.24	2,058	6.22	3.15	3,984	6.31	3.29	0.801
Age category (%)										
Below 6	6,198	6.59	1.94	2,118	6.56	1.87	4,080	6.61	1.97	0.695
6–18	6,198	13.11	2.73	2,118	13.14	2.70	4,080	13.10	2.74	0.816
19–29	6,198	12.06	2.52	2,118	12.18	2.55	4,080	12.00	2.50	0.277
30–39	6,198	16.09	3.02	2,118	16.02	2.95	4,080	16.12	3.06	0.672
40–49	6,198	13.45	2.52	2,118	13.53	2.53	4,080	13.41	2.52	0.319
50–59	6,198	13.61	2.69	2,118	13.54	2.76	4,080	13.65	2.66	0.542
60–69	6,198	13.37	3.09	2,118	13.31	3.08	4,080	13.40	3.09	0.707
Above 69	6,198	11.71	3.56	2,118	11.72	3.64	4,080	11.71	3.52	0.964
Log(population)	6,259	6.20	1.20	2,134	6.17	1.22	4,125	6.21	1.21	0.774

The table summarizes the pandemic situation at the time of the second round of the Senate elections (October 9–10, 2020), outcomes of the regional elections held 1 week earlier (October 2–3, 2020) and various socio-demographic characteristics. The *t*-test column shows *p*-values from a test of the difference in means between the constituencies renewing mandates in 2020 and the rest of the country. We approximate the reproductive number *R* using a method by an der Heiden and Hamouda (2020). Election outcomes for parliamentary parties STAN, TOP09 and KDÚ-ČSL are not reported, as these parties formed diverse electoral coalitions across regions with various local civic movements, which precludes isolating their electoral vote shares *p < 0.1, **p < 0.05, ***p < 0.01

## Empirical methodology

We use constituency-level variation in holding the 2020 Senate elections in Czechia to estimate the impact of elections on the COVID-19 pandemic. We exploit the fact that one-third of geographically scattered constituencies were assigned to renew mandates in 2020, the assignment being effectively random with respect to the initial pandemic conditions. This natural experiment allows us to estimate causal impact of elections by implementing event study design as well as simpler difference-in-differences.

### Event study design

We first outline our event study design, as it provides insights about (i) potential pre-trends, which are key for identification, and (ii) the dynamics of the treatment effect.

We develop our research hypotheses building on the insight that in the absence of mitigation policies (coordinated or self-imposed), the pandemic follows an exponential growth path that can be characterized by the initial population prevalence and the reproduction rate stemming from the inherent biological properties of the virus. Any one-time, massive (electoral) event without sufficient protective measures should increase in specific points in time the prevalence in the population. If infected cases were perfectly observable by public health authorities, the one-time event should be manifested in the data as one-time surge in new cases in the affected areas. In case of imperfect detection of new infections and variation in the incubation period, any one-time event should appear in data as a short-term acceleration in the pandemic growth, observed after an initial delay due to the incubation period. Once the increase in prevalence gets fully reflected in the data, the growth rates across the affected and non-affected areas should equalize again, although the pandemic continues growing from a higher base in the affected areas. Moreover, if a stable fraction of the new infections develops a serious condition and requires hospitalization, we should observe a temporarily linked acceleration in hospital admissions, potentially after a short delay reflecting the period between the onset of symptoms and the development of the serious condition.

Formally, we model the pandemic evolution by the following event study models:

1 $$ \frac{P_{t,m} - P_{t-n,m}}{P_{t-n,m}} = \sum\limits_{j=-J}^{K} \beta_{j} \textit{Elections}_{m} \times Day_{j} + \textbf{X}_{m,t-n}^{\prime}{\Gamma} + \lambda_{m} + \lambda_{t} + \varepsilon_{m,t} $$

2 $$ \frac{H_{t,r} - H_{t-n,r}}{H_{t-n,r}} = \sum\limits_{j=-J}^{K} \delta_{j} \textit{Elections share}_{r} \times Day_{j} + \textbf{X}_{r,t-n}^{\prime}{\Gamma} + \lambda_{r} + \lambda_{t} + \epsilon_{r,t} $$

The baseline outcomes examined in Eqs. (1) and (2) are the *n*-day growth rates in COVID-19 prevalence *P* and active hospitalizations *H*, observed in municipality *m* or commune *r* on day *t*.^8^,^9^ In Eq. (1), the independent variables of interest (event variables) are the interactions between the binary indicator for holding elections in 2020 and dummies for every date *j* in between − *J* and *K*. We set the length of the pre-treatment period to *J* = 28 days, which seems long enough to detect potential pre-trends, while setting *K* = 56 seems long enough to capture the full distributed impact of elections on new infections. To account for the non-linear nature of the pandemic evolution, we include in the model a set of time-varying control variables **X**_*m*,*t*−*n*_, which describe pandemic situation in municipalities *n* days prior to *t* (e.g. number of cumulative cases, number of active cases). Next, the model includes municipality fixed effects, *λ*_*m*_, and day fixed effects, *λ*_*t*_, to account for any time-invariant unobserved heterogeneity and country-level pandemic trend. The error term is denoted by *ε*_*m*,*t*_.

In hospitalization regression in Eq. (2), we define the event variables as a set of interactions between the share of commune population residing in voting constituencies and the dummies for every date *j* in between − *J* and *K*.^10^ The reason for this definition is that some communes include municipalities from different constituencies, some of which did not hold Senate elections in 2020. The model further includes commune fixed effects, *λ*_*r*_, day fixed effects, *λ*_*t*_, and time-varying pandemic control variables, as before. The error term is denoted by *𝜖*_*r*,*t*_.

In both specifications, we set the regressor for the first day of the second round of the Senate elections (*j* = 0) equal to zero so that all coefficients are interpreted relative to the first election day. In the estimation of Eq. (1) which uses municipality-level data, we cluster standard errors at the constituency level to allow for any unconditional heteroscedasticity as well as correlation over time in municipalities within the same constituency. In the estimation of Eq. (2) which uses hospitalization data observed at the commune level, we cluster standard errors at the commune level due to the imperfect mapping of constituencies onto communes.

### Difference-in-differences

In addition to the event study models, we implement also a set of simpler difference-in-differences which allow us to estimate the *average* acceleration in the pandemic growth in specific weeks after elections. Based on the COVID-19 incubation period and the examined 14-day pandemic growth rates, we report estimates based on data from the third week after the second electoral round (October 24–30, 2020) compared to the last week before the second round (October 3–9, 2020). Although we inspect the growth rates in the third week, the 14-day growth rates ensure that new cases and hospitalizations both from the second and third week are reflected in the inspected variable.

Formally, the difference-in-differences models can be stated as follows:

3 $$ \frac{P_{t,m} - P_{t-n,m}}{P_{t-n,m}} = \beta_{0} + \beta_{1} \textit{Elections}_{m} \times 3^{rd} week_{t} + \textbf{X}_{m,t-n}^{\prime}{\Gamma} + \lambda_{m} + \lambda_{t} + \varepsilon_{m,t} $$

4 $$ \frac{H_{t,r} - H_{t-n,r}}{H_{t-n,r}} = \delta_{0} + \delta_{1} \textit{Elections share}_{r} \times 3^{rd} week_{t} + \textbf{X}_{r,t-n}^{\prime}{\Gamma} + \lambda_{r} + \lambda_{t} + \epsilon_{r,t} $$

In Eq. (3), the variable of interest is the interaction between a dummy for elections taking place in 2020 and the indicator for days within the examined week of interest. When we examine the growth in hospitalizations in Eq. (4) using commune-level data, we define the treatment variable as the interaction between the dummy for days within the examined week after elections and the share of commune population residing in constituencies that participate in the 2020 Senate elections. Other explanatory variables and the levels of clustering remain as in the corresponding models above.

To test that elections accelerate the growth in new COVID-19 infections, we state the null hypothesis as *H*_0_ : *β*_1_ = 0 against *H*_*A*_ : *β*_1_ > 0. To test that elections at the same time lead to an acceleration in active hospitalizations, we formulate the null hypothesis as *H*_0_ : *δ*_1_ = 0 against *H*_*A*_ : *δ*_1_ > 0.

### Identification

Our models in Eqs. (1)–(4) are modifications of difference-in-differences with fixed effects, which rely on a parallel trends assumption. Put informally, identification requires that in the absence of the second round of the 2020 Senate elections, the pandemic situation would evolve along parallel paths in voting and non-voting constituencies.

The validity of the parallel trends assumption might be threatened in two ways. First, it might fail if constituencies were selected to hold elections based on their pandemic situation (or based on any long-term characteristics that determine the evolution of the pandemic). The natural experiment from the Czech Senate elections lifts this concern, as the assignment of constituencies to 2020 rotation was determined according to the election law of 1995, i.e. decades prior to the onset of the COVID-19 pandemic. Second, the parallel trends assumption might fail if public officials selectively cancelled or postponed elections in specific constituencies in the anticipation of the future development of the pandemic. In reality, we know that no constituencies cancelled or postponed the second round of the elections. Only one constituency assigned to the 2020 turn in Děčín did not hold the second round, as the winner was declared already after the first round. We do not believe this outcome might be correlated with the underlying pandemic trend that could bias our results.

We still formally check the validity of the common-trends assumption in two ways. First, in Table 1, we compared numerous observable pandemic outcomes in voting and non-voting constituencies on the election day when elections could not have yet produced any effect. We found no differences for a multitude of inspected variables. Second, our event study allows to test for differential trends in the outcomes across constituencies prior to the election day. As we show later in Section 4, there are no discernible pre-trends. Both validity checks thus strongly support our empirical strategy.

## Results

In this section, we present our estimates of the impact of large-scale, in-person elections on the COVID-19 pandemic spread in four steps. First, we examine the impact of elections on the growth in new infections. Second, we estimate the electoral effect on the growth in hospitalizations. In the third step, we inspect heterogeneity in the pandemic spread. In the fourth and final step, we shed light on the mechanism of viral spread by estimating the impact of elections on physical mobility in the election week.

### New COVID-19 cases

**Fig. 2:**
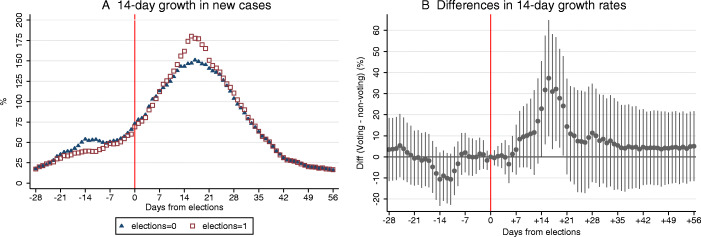
Elections and the growth in new COVID-19 cases. Panel A shows the average 14-day growth rates in new COVID-19 cases in constituencies that voted in the second round of 2020 Senate elections and those that did not, relative to the election day. Panel B reports the estimated differences in the growth rates between voting and non-voting constituencies relative to the election day obtained from Eq. (1). The panel shows 95% confidence intervals. Standard errors are clustered at the constituency level

First, we consider the effect of elections on new infections. Figure 2 reveals a rapidly accelerated growth in new COVID-19 cases in voting relatively to non-voting constituencies after the second round of the 2020 Senate elections. Panel A plots the average 14-day growth rates in new COVID-19 cases in absolute values across voting and non-voting constituencies using municipality-level data with 6,259 units observed within − 28 to + 56 days around elections (*N* = 532,015). Panel B plots coefficients *β*_*j*_ obtained from Eq. (1) which represent the estimated differences in the 14-day growth rates across voting and non-voting constituencies in every day in the inspected window around elections. The coefficients are multiplied by 100 to show percentage difference in the growth rates.

The coefficients in Panel B indicate that the 14-day growth rate in new cases started accelerating approximately 1 week after elections. This period corresponds exactly to the median incubation period for COVID-19 augmented by a lag of 2–3 days, which are likely associated with voters seeking testing and acquiring test results. The difference in the growth rates continues rising in the second week after elections and becomes significant at the 5% level 14 days after elections. The effect is most pronounced and markedly significant in the third week after elections.

Figure 8 in the Appendix shows a very similar pattern when the inspected outcome in Eq. (1) is the 7-day growth in new COVID-19 cases. The figure reveals that the 7-day growth starts accelerating 1 week after elections and is significantly faster in voting constituencies in almost the entire second week. The acceleration is slower in the third week, as the 7-day growth rate already takes the elevated growth rates on the break of the first and second weeks as the reference values.

In Table 2, we estimate the average acceleration in the 14-day growth rate in new COVID-19 cases in the third week after elections using Eq. (3). In the most parsimonious specification in column (1), we find that the 14-day growth in new cases is 24 percentage points higher in voting compared to non-voting municipalities. Relatively to the average 107% growth in all municipalities, new cases thus grow 23% faster in voting municipalities. The estimates are barely affected in column (2) where we account for (observed and unobserved) time-invariant municipality-specific factors by adding municipality fixed effects. They also remain very similar in column (3) where we control for municipal-specific time-varying pandemic situation 14 days earlier. The estimates for the interaction term in all columns are significant at least at the 5% level.^11^

**Table 2:** Growth rate in new COVID-19 cases in the third week after elections

	14-day % growth in new cases		
	(1)	(2)	(3)
Elections x third week after	24.024 **	28.000 **	24.647 **
	(11.862)	(12.564)	(10.292)
Active cases per 100k (t-14)			0.072 ***
			(0.007)
Cumulative cases per 100k (t-14)			− 0.142 ***
			(0.013)
Municipality FE	No	Yes	Yes
Day FE	Yes	Yes	Yes
Average outcome	106.53	106.53	106.53
*N* constituencies	69	69	69
*N* municipalities	6,259	6,259	6,259
*N*	87,626	87,626	87,626

The table shows difference-in-differences estimates from Eq. (3) when the examined outcome is the 14-day growth rate in new COVID-19 cases. The growth rates are measured between October 3–9 and October 24–30, 2020, i.e. within the last week before the second round of the 2020 Senate elections and in the third week after the second round. Standard errors in parentheses are clustered at the constituency level *p < 0.1, **p < 0.05, ***p < 0.01

In absolute terms, the excess number of new infections generated by elections can be calculated by multiplying (i) the estimated acceleration in the growth rate of new cases in voting relatively to non-voting constituencies in the third week after elections (which reflects the previous 14 days since the first signs of growth rate acceleration), (ii) the average prevalence of 1,231.56 cases per 100,000 people observed 1 week after elections (before the appearance of the election effect), and (iii) the population in voting constituencies. The product of these numbers corresponds to 14,858 additional cases when we binarily classify the three largest cities as treated. A more conservative estimate, which approximates the population in voting constituencies in the three largest cities by the share of voters living in their voting constituencies, would suggest excess 10,791 cases. Finally, the most conservative estimate which entirely omits the contribution of the largest cities suggests the excess of 8,692 new cases.^12^

As a robustness check, we estimate the number of excess cases also from Table 10 in the Appendix, in which we estimate the differences in 28-day growth rate in new infections across voting and non-voting constituencies 28 days after elections using simple cross-sectional OLS. If the estimates are multiplied by the population in voting constituencies and pandemic prevalence on the day of elections, we estimate excess 15,877 cases due to elections when the three largest cities are classified as treated. The similarity of the estimates with respect to the previous figures suggests that most of the additional cases arose exactly during the second and third weeks after elections.

We highlight two additional observations in Fig. 2. First, we note that the election effect fades away in the fourth and later weeks after elections. This is in line with our intuition that social interactions on the election day produce a one-time boost in the prevalence of new cases, but after it is reflected in statistics, the pandemic continues to grow at equal rate in voting and non-voting constituencies, although from an elevated base in voting constituencies. Second, we point out that there are no significant differences in the growth rate in new infections across voting and non-voting constituencies at any date prior to elections. This lack of pre-trends strongly adds credibility to the causal interpretation of our findings.

### Hospitalizations

We continue by estimating the impact of elections on the growth in hospitalizations. The advantage of this outcome is that it is far less dependent on country-specific standards in detecting and reporting COVID-19 cases. It can therefore help us validate if the acceleration in new cases is merely due to increased testing in voting constituencies.

**Fig. 3:**
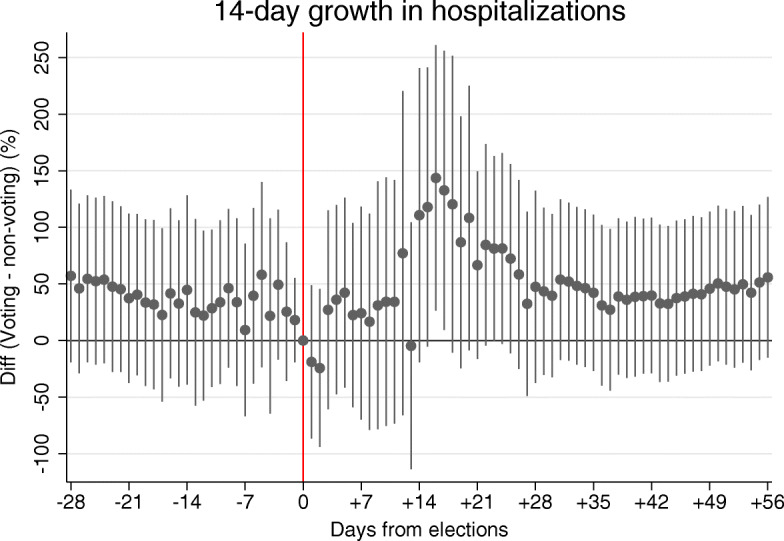
Elections and the acceleration in the growth in active hospitalizations. The figure shows the estimated coefficients for the interaction terms from Eq. (2) when the inspected outcome is the 14-day growth in active hospitalizations due to COVID-19. The figure shows 95% confidence intervals. Standard errors are clustered at the commune level

Figure 3 shows that active hospitalizations grow significantly faster in the third week after the second round of Senate elections in communes with higher shares of population from voting constituencies compared to communes with fewer eligible voters. The figure namely visualizes coefficients *δ*_*j*_ from Eq. (2) estimated using commune-level hospitalization data with 206 units within the − 28 to + 56 days frame around elections (*N* = 17,510). The coefficients are multiplied by 100 to show percentage differences in hospitalization growth rates.

A visual inspection of Fig. 3 suggests that the growth in active hospitalizations started accelerating 12–14 days after the second round of Senate elections and became significantly higher at the 5% level in communes with a higher share of eligible voters in the third week after elections. This pattern, if anything, suggests only a short delay in the growth acceleration in hospital admissions relatively to the acceleration in new infections. Figure 10 in the Appendix yet reveals that in the corresponding time period at the end of October 2020, around 50–60% of COVID-19 cases who were admitted to hospital in the Czech Republic were first tested positively for COVID-19 only after hospitalization. The figure thereby partially explains why the dates of appearance of the accelerations in new detected infections and hospitalizations are not very distant from each other.

In Table 3, we quantify the average acceleration in the hospitalization growth in the third week after elections using Eq. (4). In column (1), we report coefficients from the most parsimonious specification without commune fixed effects indicating that hospitalizations grew 62 percentage points faster in communes with 100% of population residing in voting constituencies compared to communes with zero population eligible to vote. The coefficient is significant at the 5% level. Relatively to the average 170% growth in new hospitalizations in all communes, new hospitalizations thus grow 36% faster in fully voting compared to non-voting communes. The coefficients are slightly higher in magnitude in columns (2) and (3), when we include in the model the commune fixed effects and the time-varying controls for earlier pandemic situation. The estimate in column (2) in not significant at the conventional levels (*p* = 0.106) and the estimate in column (3) is significant at the 10% level (*p* = 0.074). We argue that the standard errors are relatively high due to the relatively lower granularity of hospitalization data compared to the data on new infections observed at the municipality level.

**Table 3:** Growth in active COVID-19 hospitalizations in the third week after elections

	14-day % growth in hospitalizations		
	(1)	(2)	(3)
Elections share x third week after	61.683 **	74.255	78.709 *
	(31.030)	(45.715)	(43.852)
Active cases per 100k (t-14)			0.069
			(0.105)
Cumulative cases per 100k (t-14)			− 0.336 ***
			(0.075)
Commune FE	No	Yes	Yes
Day FE	Yes	Yes	Yes
Average outcome	169.64	169.64	169.64
*N* communes	206	206	206
*N*	2,884	2,884	2,884

The table shows difference-in-differences estimates from Eq. (4) when the examined outcome is the 14-day growth in active hospitalizations. The growth rates are measured between October 3–9 and October 24–30, 2020, i.e. within the last week before the second round of the 2020 Senate elections and in the third week after the second round. Standard errors in parentheses are clustered at the commune level *p < 0.1, **p < 0.05, ***p < 0.01

If we take the results presented so far together, we note that the acceleration in hospitalization (around 36.4–46.4% relatively to the average growth rate) is somewhat higher than the acceleration in new cases (around 22.5–26.3%). This could be expected when citizens are reluctant to get tested or when testing facilities are overloaded and citizens are not tested until the disease progresses into a more severe phase requiring hospitalization. In both scenarios, the temporarily interlinked nature of the two accelerations rules out that estimated effect of elections on viral spread would be merely due to increased testing in voting constituencies. In Fig. 11 in the Appendix, we provide additional evidence against this hypothesis by inspecting the differences in average 7-day positivity rates across voting and non-voting constituencies using event study specification from Eq. (2) and commune-level data. The figure indicates the positivity becomes around 1 percentage point lower in constituencies with 100% of voting population in the second week after elections compared to constituencies with zero eligible voters. The effect is not statistically significant at the conventional levels. It is also not large in magnitude especially when expressed relatively to the average positivity of 32.8% observed in the second week after elections. In sum, our evidence suggests that changes in the intensity of testing likely play a limited role in the observed pandemic acceleration.

### Heterogeneity

Next, we examine heterogeneity in the pandemic acceleration with respect to the observable characteristics of the infected cases and municipal population.

**Fig. 4:**
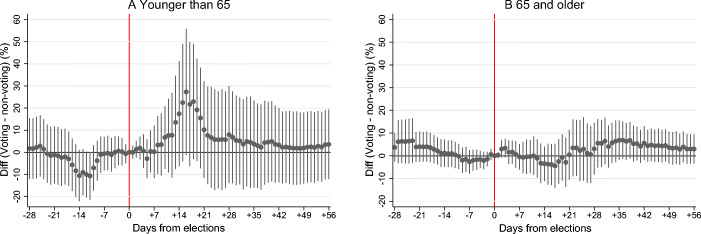
Heterogeneity in the acceleration in new COVID-19 cases with respect to age. Panels A and B show heterogeneity in the estimated acceleration in 14-day growth rates in new COVID-19 cases in the population younger and older than 65, respectively, across voting and non-voting constituencies, relative to the election day. The figures show 95% confidence intervals. Standard errors are clustered at the constituency level

We start by examining pandemic acceleration in new COVID-19 infections separately in population younger and older than 65. In panels A and B in Fig. 4, we plot coefficients *β*_*j*_ obtained from Eq. (1) when the 14-day growth rate in new infections is calculated only using cases younger and older than 65, respectively. In Panel A for cases younger than 65, we depict the differences in the pandemic growth rates across voting and non-voting municipalities that are very similar to what we observe in Fig. 2 for the whole population. Approximately 1 week after the elections, the 14-day growth rate in new cases starts accelerating in voting relatively to non-voting municipalities. The acceleration is discernible during the second and third weeks after elections, and later the election effect fades away. Statistically, the coefficients are significant at the 10% level around the peak of the acceleration. In Panel B, we observe a very different pattern for the population older than 65. There is essentially no discernible acceleration in the pandemic growth in voting relatively to non-voting constituencies within the entire examined period of 2 months after the elections.

We interpret the differential acceleration as evidence consistent with strategic risk-avoidance by older voters (Dave et al. 2020b), for whom COVID-19 represents a major risk of hospitalization and dying (Williamson et al. 2020). In theory, the reasons for the differential impact might be that either older cohorts are more cautious in taking preventive measures and following social distancing protocols or they are simply more likely to abstain from elections. Since elections are anonymous and exit polls were not conducted, we do not observe turnout by age groups. In Fig. 12 in the Appendix we however plot the associations between municipal share of population older than 65 and total turnout in 2016 and 2020 regional elections, which were held together with the first rounds of the Senate elections.^13^ We find that in the 2016 elections, a 1% higher share of population above 65 was associated with 0.511% higher turnout. In the 2020 regional elections, held 1 week before our natural experiment, the estimated association was four times lower in magnitude and insignificant at the conventional levels if one accounts for municipal population size. The weaker association in 2020 together with the pandemic acceleration absent in population above 65 point towards increased absenteeism in elections by older cohorts.

**Fig. 5:**
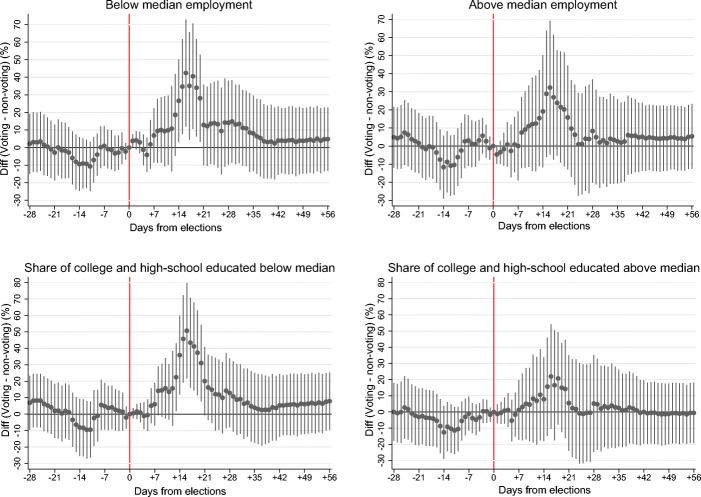
Heterogeneity in the acceleration in new COVID-19 cases with respect to municipal employment and education. The upper two panels estimate Eq. (1) for municipalities divided with respect to the median employment. The bottom two panels estimate the same specification for municipalities divided with respect to the median share of individuals with at least secondary education. The figures show 95% confidence intervals. Standard errors are clustered at the constituency level

**Fig. 6:**
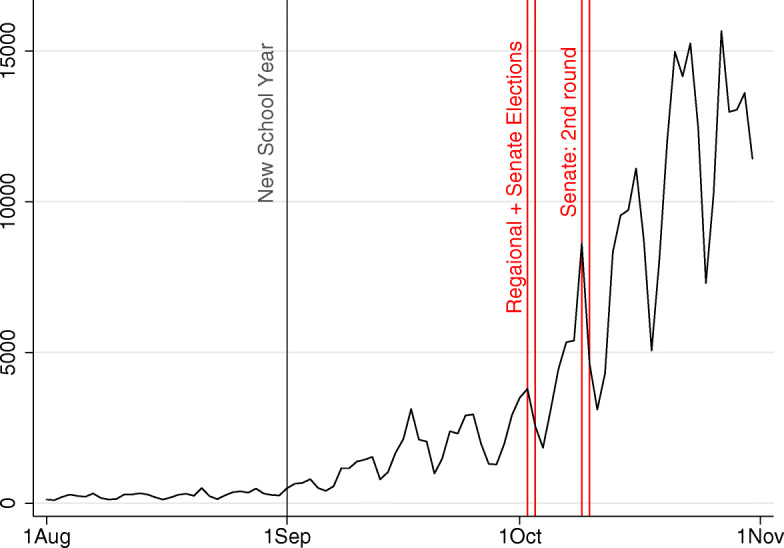
Daily incidence of detected COVID-19 cases. The figure plots the daily incidence of new COVID-19 cases in the Czech Republic since August 2020. The vertical lines mark the beginning of the school year for elementary schools and high schools and the dates of the 1st and the 2nd round of the 2020 Senate elections In the Czech Republic (two days in each round)

**Fig. 7:**
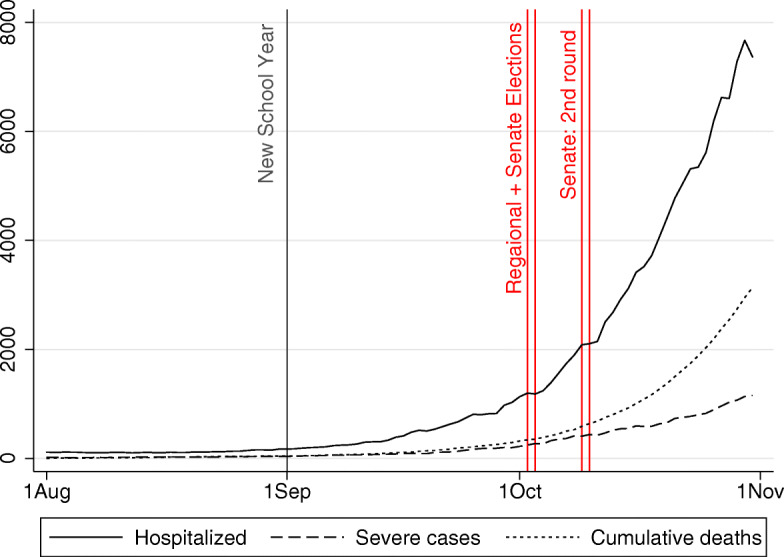
Hospitalizations and cumulative deaths officially due to COVID-19. The figure shows active hospitalizations and cumulative deaths due to COVID-19 in the Czech Republic since August 1, 2020. During the first wave and up until August 2020 approximately 380 people died in Czechia due to COVID-19 according to the official counts

**Fig. 8:**
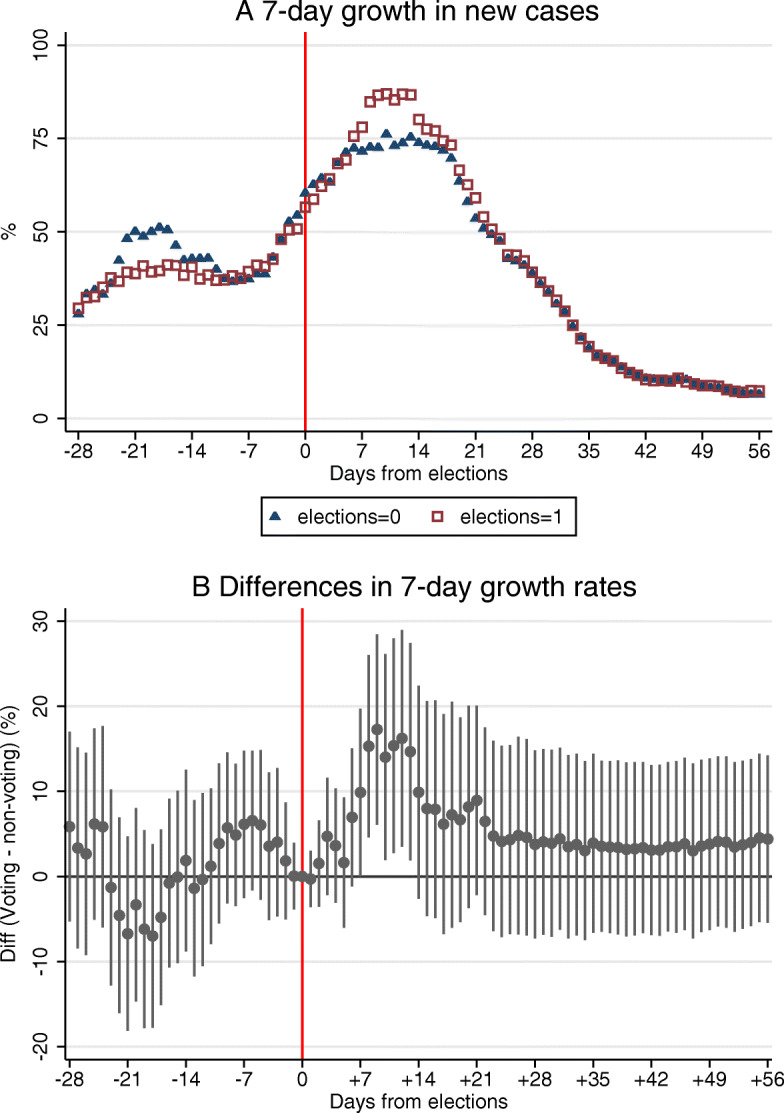
Elections and the growth in new COVID-19 cases. Panel A shows the average 7-day growth rates in new COVID-19 cases in constituencies that voted in the second round of 2020 Senate elections and those that did not, relative to the election day. Event study graph in panel B shows the estimated differences in the 7-day growth rates across voting and non-voting constituencies, relative to the election day, estimated by Eq. (1). The panel shows 95% confidence intervals. Standard errors are clustered at the constituency level

**Fig. 10:**
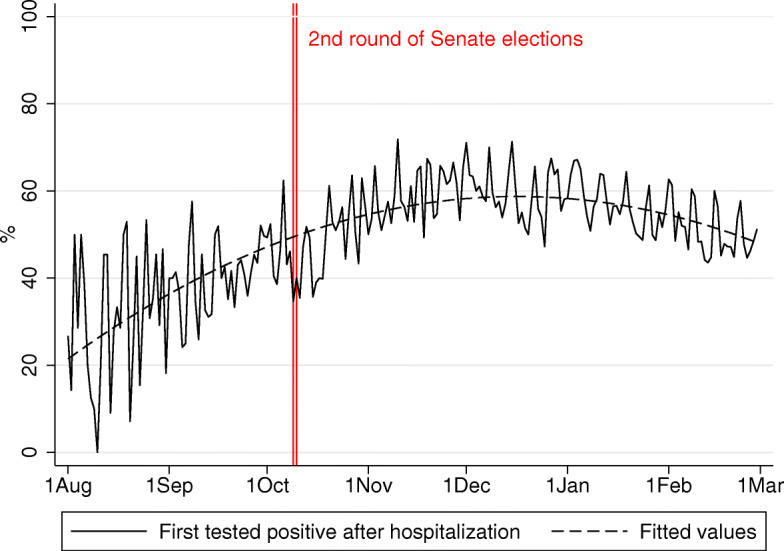
The share of hospital admissions without a prior positive COVID-19 test. Share of hospitalized COVID-19 patients who were first tested positive after being admitted to hospital. The figure is plotted using data between August 1, 2020, and March 1, 2021. Source: The Ministry of Health of the Czech Republic and the Institute of Health Information and Statistics

**Fig. 11:**
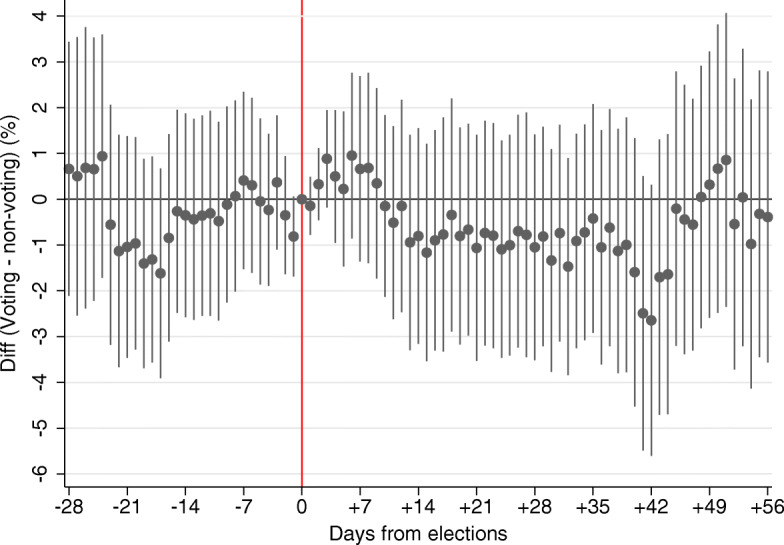
The rate of positivity of COVID-19 tests. The event study graph shows the estimated coefficients for the interaction terms from Eq. (2) when the inspected outcome is the 7-day positivity rate of COVID-19 PCR tests. The average positivity in the second week after elections was 32.77%. The figure shows 95% confidence intervals. Standard errors are clustered at the commune level

**Fig. 12:**
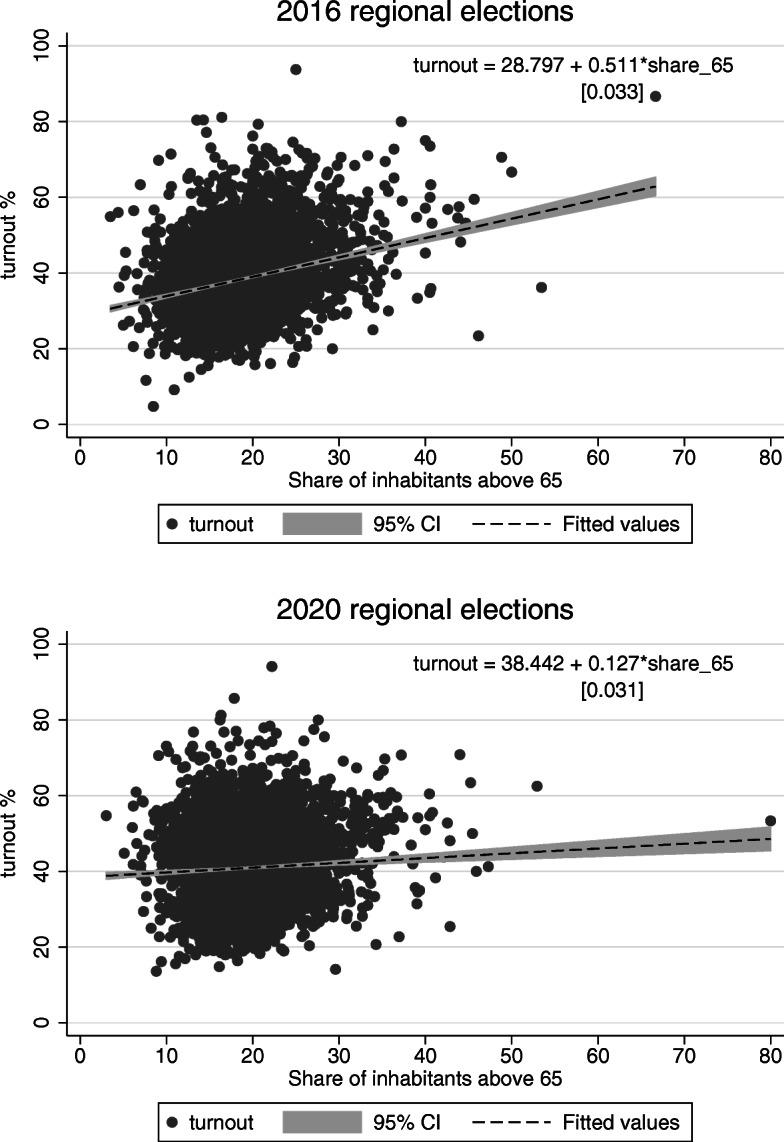
The associations between the share of residents above 65 and voter turnout. The panels in figure show statistical associations between the share of municipal residents above 65 and voter turnout in 2016 and 2020 regional elections respectively. The dashed lines are predicted values from a bi-variate OLS regression with the estimated regression coefficients and the corresponding standard errors reported in the upper right corner. The panels show 95% confidence intervals around the predicted regression lines

In Fig. 5, we continue examining heterogeneity in the pandemic acceleration due to elections with respect to socio-economic conditions in municipalities. In particular, we proceed by dividing the sample of all municipalities into halves according to the median values of municipal employment and the median share of individuals with at least secondary education, respectively. Then, we estimate Eq. (1) using each of the reduced samples and the 14-day growth in new infections as the outcome variable.

In all panels in Fig. 5, we observe a discernible acceleration in the pandemic growth rate in voting compared to non-voting constituencies peaking in around the third week after elections. We note that the acceleration is significant at the 5% level only in municipalities with below-median employment and below-median share of individuals with at least secondary education. When we test the equality of the acceleration across municipalities with below- and above-median levels of employment, we however do not find statistically significant differences. On the other hand, we find the acceleration significantly higher in municipalities with below-median share of individuals with at least secondary education compared to municipalities above the median. We interpret our results as consistent with the literature suggesting that socio-economic factors play an important role for the speed of the pandemic spread and its mitigation. In this literature, for example, Wright et al. (2020) show that regions with higher economic endowment are more likely to comply with anti-pandemic measures.^14^

### Physical mobility and social interactions

Finally, we examine the mechanism of viral spread by asking if elections are associated with spikes in physical mobility, and if so, what specific mode of social interactions might have contributed to faster pandemic spread on the electoral days.

**Fig. 14:**
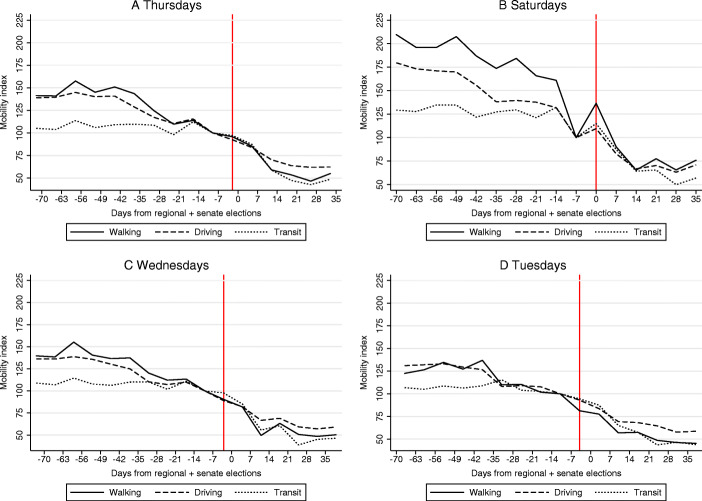
Apple mobility indices, week-by-week, by the day of the week. The figure shows trends in the Apple mobility indices for various days of the week relative to the first round of the 2020 regional elections. In panel A, the vertical line marks the last Thursday before the elections. The indices are normalized to 100 on Thursday 9 days before elections. In panel B, the vertical line marks the electoral Saturday. The mobility indices are normalized to 100 on the last Saturday (7 days) before elections. In panel C, the vertical line marks the last Wednesday before the elections. The mobility indices are normalized to 100 on Wednesday 10 days before elections. In panel D, the vertical line marks the last Tuesday before the elections. The mobility indices are normalized to 100 on Tuesday 11 days before elections

In Fig. 14 in the Appendix, we start the analysis by visualizing the association between the first round of Senate elections and country-level mobility indices from Apple. Panels A, C and D namely plot mobility indices for Thursdays, Wednesdays and Tuesdays (non-electoral days), respectively, in a range of − 10/+ 5 weeks around the elections. Panel B plots the mobility indices for Saturdays (electoral day) within the same time frame. In all panels, the figure shows a generally declining trend in all examined types of mobility (walking, driving, transit), consistently with the expectation that people were continuously limiting mobility in the face of the progressing pandemic and government restrictions. At the same time, Panel B shows a pronounced spike in mobility on the electoral Saturday. The magnitude of the spike should be however interpreted as suggestive, as any day-specific shocks, such as favourable weather conditions, might have elevated mobility on the electoral day. At the same time, the data rely on users of Apple devices, which might be more strongly represented in larger cities with higher turnout.

In Table 4, we provide evidence from a representative survey “Life during the pandemic” which has been following a panel of Czech households since mid-March 2020. Respondents from across different districts were bi-weekly asked about the frequency of various social activities which they had participated in each of the previous two weeks (such as in-person shopping, family visits, visits to restaurants and pubs, group holidays, attendance in large public events). The table shows estimates from random effects multinomial ordered logistic regressions, in which the outcomes are categorical variables representing the frequency of particular activities. In fashion of Eq. (4), the independent variable of interest is the interaction between the dummy for the electoral week and the share of the district population living in voting constituencies.

**Table 4:** Physical mobility and social activities in the electoral week, results from a representative survey

	Never	1-2x	≥ 3x	Never	1-2x	≥ 3x	Never	1-2x	≥ 3x
	Use of public transport			Shopping			Family visits		
Elections share x election week	− 0.061*	0.058*	0.004	− 0.003	0.000	0.002	− 0.029	0.028	0.001
	(0.036)	(0.034)	(0.002)	(0.008)	(0.002)	(0.007)	(0.025)	(0.024)	(0.001)
Share of responses	0.56	0.27	0.16	0.18	0.66	0.16	0.38	0.57	0.05
	Visits to restaurants & pubs			Group holidays & trips			Large public events		
Elections share x election week	− 0.013	0.012	0.000	− 0.006	0.006	0.000	− 0.000	0.000	0.000
	(0.037)	(0.036)	(0.001)	(0.019)	(0.018)	(0.001)	(0.004)	(0.004)	(0.000)
Share of responses	0.55	0.38	0.07	0.83	0.16	0.01	0.92	0.08	0.00

The table shows marginal effects from random effects multinomial ordered logistic regressions based on weekly data from a representative panel survey from August 3–October 11, 2020. Marginal effects are calculated at means of the explanatory variables assuming respondent random effects equal zero. All regressions include week fixed effects. *N* = 22, 078. Standard errors in parentheses are clustered at the district level

*p < 0.1, **p < 0.05, ***p < 0.01

The table indicates that the probability that respondents travelled at least once by crowded public transport increased 6.1 percentage points (10.9%) in districts with 100% of population from voting constituencies in the week of the second electoral round compared to districts with no such population. The estimate is significant at the 10% level. It is also quantitatively feasible given the turnout of around 16.74% in the second electoral round. At the same time, we estimate that none of the other surveyed activities were statistically more likely to be carried out by respondents in the election week in districts with higher shares of population from voting constituencies.

The null results for additional social activities are supported by Table 5, in which we use Google mobility reports to examine the effect of elections on physical mobility at six different general types of locations (retail and recreation facilities, groceries and pharmacies, parks, transit stations, workplaces, and residential areas). Using a variant of Eq. (4), we find that elections are not significantly associated with higher mobility at any of these locations. On the other hand, they are significantly linked with a shorter length of stays of the tracked devices at residential locations on the electoral day (column 6). The effect is significant at the 5% level.

**Table 5:** Google mobility trends

	Retail &	Grocery &		Transit		
	recreation	pharmacy	Parks	stations	Work	Residential
	(1)	(2)	(3)	(4)	(5)	(6)
Election share x election week	− 1.425	− 1.018	4.839	− 1.670	1.056	− 0.478**
	(2.264)	(2.038)	(6.434)	(3.541)	(0.897)	(0.224)
Election week	− 7.375***	− 1.422	22.361***	5.512***	3.098***	0.217
	(1.448)	(1.153)	(4.046)	(1.915)	(0.611)	(0.136)
Average outcome	− 14.18	10.96	18.73	− 6.66	− 0.19	3.25
*N*	148	141	107	122	154	138

The table reports difference-in-differences estimates for various categories of Google mobility indices measured on October 10, 2020 and September 26, 2020 i.e. during the second round of the 2020 Senate elections and 2 weeks earlier. All regressions include district fixed effects. Standard errors in parentheses are clustered at the district level *p < 0.1, **p < 0.05, ***p < 0.01

In sum, our estimates suggest that in-person elections are linked with elevated mobility, which does not seem to be related with higher social interactions in any of the examined locations outside polling stations. One should yet remain cautious in interpretation, as it is possible that the examined survey omits an important category of activities or that Google does not track a category of locations that were key to viral spread. In addition, it is important to remember that many of primary infections from the election days produce secondary and tertiary cases in voters’ households and workplaces, contributing to the total acceleration in new cases due to in-person voting.

## Conclusion

In this paper, we estimate the causal impact of holding large-scale, in-person elections on the viral spread of COVID-19. We avoid strong assumptions about voter turnout and pre-electoral pandemic trajectories by exploiting the natural experiment from the second round of the 2020 Senate elections in the Czech Republic (held on October 9–10), which renewed mandates in one-third of constituencies pre-selected according to a 25-year-old rotation rule.

Using event study design, we estimate that the growth in new COVID-19 cases is significantly higher in voting compared to non-voting constituencies in the second and third weeks after elections. A significant, temporarily linked acceleration in hospital admissions and essentially no changes in test positivity rates suggest that our estimates cannot be merely due to increased testing. We find the acceleration to be absent in the population above 65 and pronounced in municipalities with below-median share of inhabitants with at least secondary education. Compared to non-pharmaceutical interventions evaluated by Brauner et al. (2021), our estimates correspond approximately to the growth in the reproduction rate achieved by re-opening most of the non-essential face-to-face businesses for the corresponding period of time (1–2 days).

For correct interpretation of our estimates, it is important to consider that our empirical method implicitly relies on the assumption of no spillovers between voting and non-voting constituencies. This assumption is likely valid in the initial days and weeks after the elections, but its credibility continuously diminishes over time, for example, due to spacial flows of labour force. Even though it is beyond the scope of this paper to quantify the empirical relevance of spatial spillovers, it is worth noting that such spillovers in theory eliminate the differences in pandemic growth rates between voting and non-voting constituencies. Spatial spillovers thus plausibly attenuate our estimates. Similarly, our estimates represent a lower bound of the contagion effect if citizens in voting constituencies reduce their economic activity immediately after elections to compensate for the increased risk of contagion during elections.

The next natural question is that of external validity. We recognize that there is heterogeneity in how citizens in different countries perceive their civic duty to go to vote, how easy and trustworthy it is for citizens to participate in postal elections and how strictly they comply with anti-pandemic interventions. Certain attributes of the Czech electoral system should be taken into consideration. For instance, the country does not allow other than in-person voting and turnout is generally lower than in many more developed democracies. In this context, we note that the estimated pandemic acceleration was considerably high, even though in-person turnout in the examined elections was only 16.7%. We believe that additional physical mobility generated by higher turnouts in other countries with similar preventive measures and compliance can only increase the pandemic potential of the election days, especially in the presence of more infections variants of the virus. Similarly, one could expect the viral spread to be higher in countries with lower compliance with anti-pandemic measures if this is dictated by worse economic conditions (Wright et al. 2020).

From a long-term perspective, our evidence suggests that large-scale, in-person (electoral) events can be an important accelerator of the spread of viral diseases, providing a credible resolution for the literature on this question. Additionally, our paper provides evidence consistent with strategic risk-avoidance by older voters (Dave et al. 2020b), which may be especially problematic in the context of democratic elections if the risk of contagion disturbs equal electoral franchise.

Regarding policy implications, democracies considering to mitigate the pandemic impact of large-scale, electoral events face a limited set of options. First, if viral spread occurs mostly at polling places, countries can consider strict anti-pandemic measures on electoral premises, including checks on adequate ventilation, physical distancing and the community use of well-fitting masks (CDC 2021). This option however does not prevent increased concentrations of people and does not address restrictions on equal electoral franchise, as voters who are at the greatest risk might increasingly abstain from elections even under strict anti-pandemic measures. The second option is to consider postponing in-person elections until viral spread is controlled and health risks are minimized. This option addresses the concern of unequal franchise and reduces viral transmission even if it does not occur primarily at polling places. The main disadvantage is that postponing of elections interferes with the electoral accountability of previously elected politicians. The last option is to facilitate voting by other than in-person methods (e.g. by post or online). Although our study does not evaluate various aspects of absentee voting, we note that it does not interfere with electoral accountability and helps restoring equal franchise. In the US setting, the universal vote-by-mail option has been shown not to favour particular parties (Thompson et al. 2020), even though the US electorate has become increasingly polarized about its use during the COVID-19 crisis (Lockhart et al. 2020). Compared to in-person voting, both postal and online methods require fewer human interactions, which can help reducing the pandemic spread. Positive examples from countries such as Switzerland, where postal voting is the dominant method of voting, illustrate that mail-in voting can be organized robustly without generating doubts about potential voter fraud or significant delays in counting the ballots.

## Figures and Tables

**Fig. 9:**
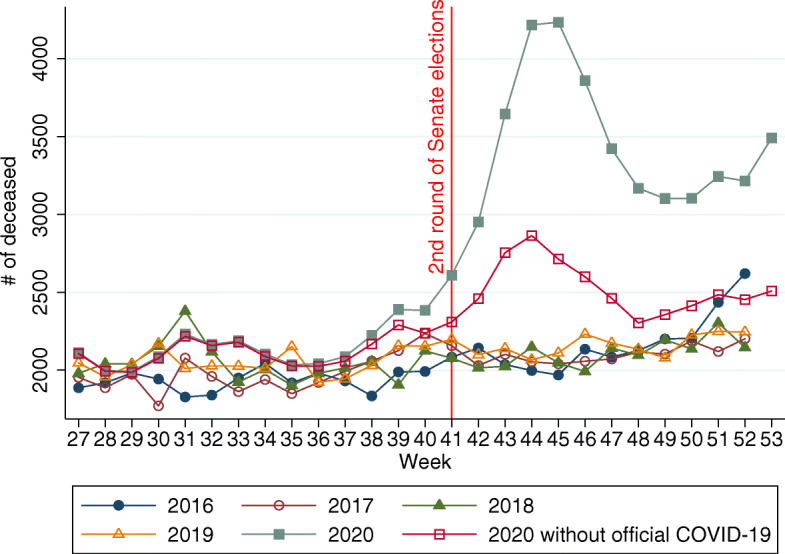
All-cause mortality by calendar weeks: 2016–2020. The figure plots weekly all-cause mortality in the Czech Republic in weeks 27–53 in years 2016–2020. For 2020, we plot both the total all-cause mortality and the total mortality net of the official COVID-19 cases. The vertical line marks the week of the second round of the 2020 Senate elections

**Fig. 13:**
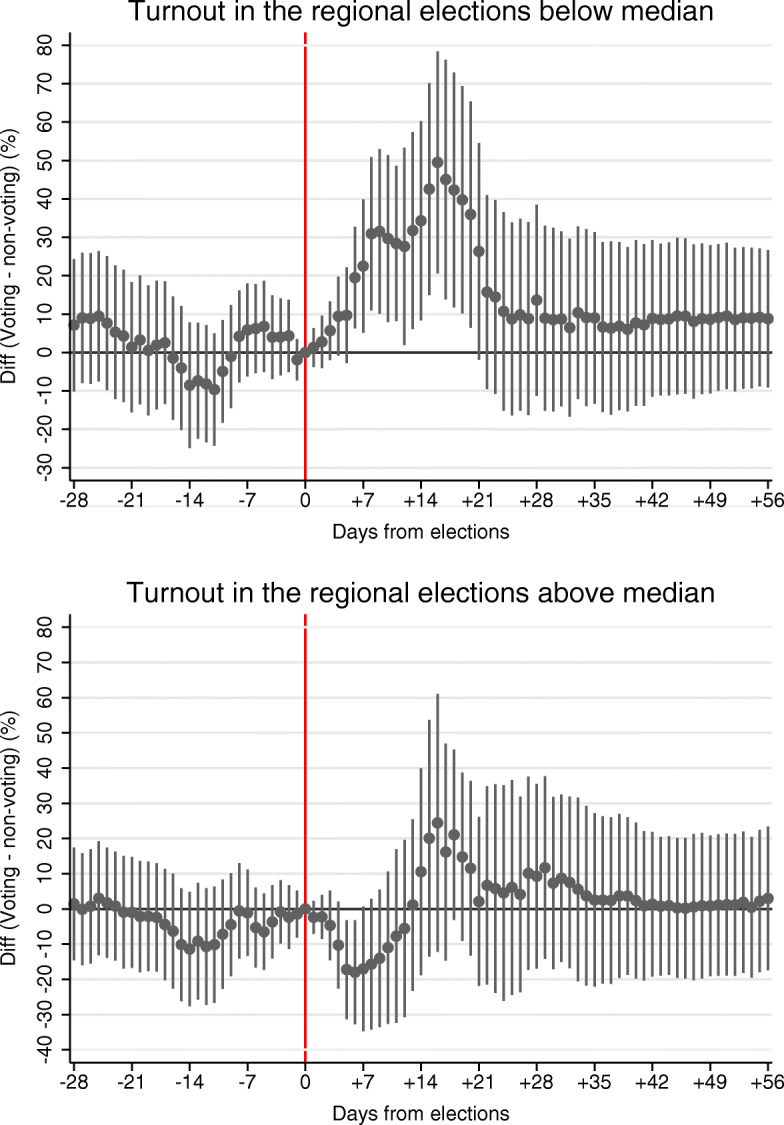
Heterogeneity in the growth rate acceleration in new COVID-19 cases with respect to the turnout in the 2020 regional elections. The figure shows differences in the 14-day growth rates in new COVID-19 cases across constituencies that voted in the 2020 Senate elections and those that did not, relative to the election day, across municipalities divided into halves with respect to the median turnout in the 2020 regional elections (held 1 week before the second round of the Senate elections). Panel A shows the estimated differences in the examined growth rates for municipalities with below-median voter turnout. Panel B shows the estimated differences for municipalities with above-median turnout. Both panels show 95% confidence intervals. Standard errors in both panels are clustered at the constituency level

## Data Availability

The datasets used to produce the main results are available at public repositories, as specified in Table 8 in the Appendix.

## Appendix

**Table 6:** Partisan positions towards anti-COVID-19 interventions

**1. ANO 2011**
*Ideology:* A conservative, populistic party, initially built upon anti-corruption rhetoric and calls for higher public sector efficiency. **Leading the government.**
*Electorate:* voters of all ages, dominant among voters above 45, people with high school or elementary education, voters from middle-sized towns and rural areas
*Vote share in the 2017 national elections:* 29.6% (78/200 seats)
*Position towards anti-Covid-19 interventions:* Prior to 2020 regional and Senate elections, the party was reluctant to impose strict anti-Covid interventions, including those with low economic costs, such as mandatory face masks. After elections, under the weight of the situation, the party left most of the decision making to epidemiologists and other academic experts.
**2. Civic Democratic Party (ODS)**
*Ideology:* A right-wing, conservative party promoting liberal economy policies.**In opposition.**
*Electorate:* business people, self-employed, urban dwellers
*Vote share in the 2017 national elections:* 11.3% (25/200 seats)
*Position towards anti-Covid-19 interventions:* In summer 2020, the party opposed mandatory face masks. After the elections, it criticized chaotic governmental management of the pandemic.
**3. Czech Pirate Party**
*Ideology:* A liberal, center-left party. **In opposition.**
*Electorate:* students and younger generations, urban dwellers
*Vote share in the 2017 national elections:* 10.8% (22/200 seats)
*Position towards anti-Covid-19 interventions:* Prior to summer 2020, the party suggested investments into public testing and tracing capacities and requested policy-making based on consultations with academia. The party however did not have representatives in the Central Crisis Staff in autumn 2020.
**4. Freedom and Direct Democracy (SPD)**
*Ideology:* A far-right, nationalistic, anti-immigrant party calling for more frequent use of direct referenda and revocability of politicians. **In opposition.**
*Electorate:* voters with elementary education, rural dwellers
*Vote share in the 2017 national elections:* 10.6% (22/200 seats)
*Position towards anti-Covid-19 interventions:* The party, perhaps surprisingly, remained mostly silent during the autumn wave of the 2020 pandemic and did not bid its voters to boycott governmental interventions.
**5. Communist Party of Bohemia and Moravia (KSČM)**
*Ideology:* A far-left party advocating strong state interventions and higher taxes for the rich.
**In silent coalition with the government.**
*Electorate:* pensioners, manual workers, supporters of strong welfare state
*Vote share in the 2017 national elections:* 7.8% (15/200 seats)
*Position towards anti-Covid-19 interventions:* The party mostly did not feature in public discussions regarding the interventions.
**6. Czech Social Democratic Party (ČSSD)**
*Ideology*: A traditional, left-wing party supporting socially oriented policies. **In government.**
*Electorate:* manual workers, state employees, pensioners, voters from industrial regions
*Vote share in the 2017 national elections:* 7.3% (15/200 seats)
*Position towards anti-Covid-19 interventions:* In autumn 2020, the party suggested earlier interventions, but remained mostly ignored due to its low bargaining power in the government.
**7. Christian Democratic Union – Czechoslovak People’s Party (KDU-ČSL)**
*Ideology:* A centrist, conservative party with Christian-social orientation. **In opposition.**
*Electorate*: Catholics, pensioners, voters in the region of southern Moravia
*Vote share in the 2017 national elections:* 5.8% (10/200 seats)
*Position towards anti-Covid-19 interventions:* The party mostly did not feature in public discussions regarding governmental interventions.
**8. TOP 09**
*Ideology:* A right-wing, conservative party advocating fiscal prudency and little state interventions. **In opposition.**
*Electorate:* voters in the capital city of Prague, business people, wealthier voters
*Vote share in the 2017 national elections:* 5.3 % (7/200 seats)
*Position towards anti-Covid-19 interventions:* Some party experts strongly opposed governmental interventions, but later disappeared from public discussions.
**9. Mayors and Independents (STAN)**
*Ideology:* A liberal, centrist party drafting candidates mostly from regional and local politics.
**In opposition.**
*Electorate:* voters of all ages, voters with higher education
*Vote share in the 2017 national elections:* 5.2% (6/200 seats)
*Position towards anti-Covid-19 interventions:* Similarly as the Pirate party, Mayors and Independents requested higher investments into the testing and tracing capacities. Their voice remained mostly unheard.

**Table 7:** Timeline of government interventions

Date	New restrictions, most important events	14-day incidence
		per 100, 000
Situation as of	–Face masks mandatory in the subway of the capital of Prague	41.5
31.08.2020	–Recommendations to keep physical distance and perform frequent hand hygiene	
01.09.2020	– **Start of the school year**	43.5
	–Mandatory face mask in public transport, public institutions, retirement homes and health facilities	
10.09.2020	–Face masks mandatory indoors, except for classrooms and private housing	87.7
18.09.2020	–Face masks mandatory in classrooms for teachers and children above 10	178.5
21.09.2020	–Resignation of the Minister of Health	207.0
	–**Formal establishment of the Central Crisis Staff**	
24.09.2020	–Closing time at restaurants set at 10:00 pm	218.5
	–Audience in outdoor public events and sports matches limited to 2000 people if these have assigned seats	
	–Attendance in outdoor public events limited to 50 if no seats are assigned	
	–Attendance in indoors public events limited to 10 if no seats are assigned	
02.-03.10.2020	–**Regional and Senate elections - 1**^**st**^ **round**	**298.6**
05.10.2020	–**Declared State of Emergency**	326.9
	–Sport matches played without audience	
	–Singing forbidden in theatre plays, operas and musicals	
	–At most 6 people can sit at one table in restaurant	
09.-10.10.2020	–**Senate elections - 2**^**nd**^ **round**	**251.3**
	–Closure of indoor sport facilities, outdoor sport restricted to 20 people	
	–Restrictions on physical mobility in shopping malls	
	–Closing time in restaurants set at the latest at 8:00 pm	
	–At most 4 people can sit at one table	
10.10.2020	– **Distance schooling introduced at universities and selected high school**	521.6
	–Rotation systems introduced at elementary scholl for children above 10	
	–Restrictions on public gatherings (10 indoors and 20 outdoors)	
	–Closure of cinemas, theatres, castles and ZOOs	
	–Public institutions open 2 days in week. 5 hours per day	
	–Forbidden visits in elderly homes and hospital	
14.10.2020	–**Full closure of elementary school’s and high school’s**	643.4
	–Dining fully forbidden inside of restaurants	
10.10.2020	–Face masks mandatory everwhere outdoors within town limits or if 2-meter perimeter cannot be kept	905.1
22.10.2020	–Closure of retail shops (except for food stores, drugstores and pharmacies) and personal beauty services	1148.5
	–Outdoor physical mobility limited (with exceptions for travel to work, to do shopping, to visit doctor or family)	
	–Public institutions attend only emergency cases	
26.10.2020	–**Curfew between 9:00 p.m. and 5:00 a.m.**	1379.8

**Table 8:** Data sources and descriptions

Data set	Variables description	Observation level, # of units	Periodicity, time span	Providers:	Url:
COVID-19 incidence & prevalence	New Covid-19 cases, active cases, cumulative Covid-19 cases, reproductive number R, separately for population ≥ 65	6,259 municipalities	Daily, Mar 1, 2020–Dec 4, 2020	Ministry of Health, Institute of Health Information and Statistics	https://onemocneni-aktualne.mzcr.cz/api/v2/covid-19
COVID-19 hospitalizations & positivity	Hospitalizations, PCR positivity rates	206 communes	Daily, Mar 1, 2020–Dec 4, 2020	Ministry of Health, Institute of Health Information and Statistics	https://onemocneni-aktualne.mzcr.cz/api/v2/covid-19
Regional and Senate elections	Vote shares by party lists, electorate size, turnout, constituency ids	6,259 municipalities	Cross-sections, Oct 2–3, 2020, Oct 9–10, 2020	Czech Statistical Office	https://www.volby.cz/opendata/opendata.htm
2011 population and housing census	Population data on socio- economic status, age and education level	6,259 municipalities	Cross-section, 2011	Czech Statistical Office	https://www.czso.cz/csu/czso/csu_a_uzemne_analyticke_podklady
PAQ panel survey	Representative survey on physical mobility and social interactions	$\sim $ 2,200 respondents in 76 districts and Prague	Weekly, Aug 3– Oct 11, 2020	PAQ research	Publicly unavailable
Google mobility	Physical mobility indices, by location type	76 districts and Prague	Daily, Feb 15–Nov 1, 2020	Google	https://www.google.com/covid19/mobility/
Apple mobility	Physical mobility indices, by transportation mode	Country level	Daily, Jan 13–Nov 1, 2020	Apple	https://covid19.apple.com/mobility

**Table 9:** Questions in the PAQ “Life during the pandemics” survey

	Please, indicate if you personally have participated in between	I have not	Yes,	Yes,
	*date1* and *date2* in the following activities:	participated in	personally	personally
	*(choose one answer in each row)*	this activity	1-2x	more frequently
1.	Travel by full public transport, train or bus	.	.	.
2.	Shopping (or a visit to a bank / post office) with an elevated presence of people	.	.	.
3.	Visit to family or friends (at their or your place)	.	.	.
4.	Visit to a restaurant or a bar	.	.	.
5.	Group holiday or a trip with numerous attendants	.	.	.
6.	Visit to a public sports event, concert or a cultural event (with 50 to 500 attendants)	.	.	.

**Table 10:** The overall effect of elections: Cross-sectional OLS regressions

	28-day % growth in new cases				
	(1)	(2)	(3)	(4)	(5)
Elections	43.939	44.346**	46.604**	41.499**	42.314**
	(29.666)	(21.244)	(21.737)	(19.842)	(19.864)
Active cases per 100k (t-28)				0.020	0.020
				(0.015)	(0.015)
Cumulative cases per 100k (t-28)				− 0.100***	− 0.100***
				(0.015)	(0.015)
Log(population)			98.970***	104.667***	105.787***
			(8.081)	(7.883)	(7.885)
Region FE	No	Yes	Yes	Yes	Yes
					Without Prague,
					Brno and Ostrava
Average outcome	366.60	366.60	366.60	366.60	366.68
*N* constituencies	69	69	69	69	67
*N* municipalities	6,259	6,259	6,259	6,259	6,256

The table reports estimates from cross-sectional OLS regressions for the 28-day growth rate in new COVID-19 cases measured 28 days after the second round of the 2020 Senate elections, regressed on a binary indicator for elections taking place in municipalities in 2020 and covariates controlling for pandemic situation in municipalities on the election day. Standard errors in parentheses are clustered at the constituency level * *p* < 0.1, ***p* < 0.05, ****p* < 0.01

## Footnotes

1 Similar concerns apply to other political events, such as electoral rallies. Empirical evidence in this context is however also inconclusive. For example, Dave et al. (2020a) use synthetic control methods to find little evidence that a highly publicized Trump campaign rally at Tulsa, Oklahoma led to an increase in new COVID-19 cases or deaths. In contrast, Bernheim et al. (2020) aggregate 18 Trump campaign events and find sizable effects, estimating 30,000 additional cases and 700 deaths as a result of these rallies. In their additional analysis, the authors find that COVID-19 outcomes at the examined events are highly variable, which helps to explain why a single event like at Tulsa might not be representative.

2 See Blais (2006) and Geys (2006) for comprehensive reviews of literature on the political and socio-economic determinants of voter turnout.

3 There is practically no relation between the regional elections and the first round of the Senate elections, apart from the fact that they are held on the same days in the same voting rooms. Voters cast ballots for separate and independent candidates (and candidate lists) into separate ballot boxes. The main motivation for the joint organization of elections is to save on the remuneration of electoral committees and to increase voter turnout.

4 Table 8 in the Appendix describes the observation level, periodicity, time span and sources for all data sets used in our analysis.

5 In response to the pandemic, the Czech Republic constructed two temporary field hospitals in Fall 2020 in Prague and Brno (the two largest cities). Nevertheless, neither of these hospitals had been ever occupied by patients. The hospital in Prague was deconstructed amid the temporary drop in cases in January 2021. The hospital in Brno likewise remained vacant, but was later used as a vaccination center.

6 Given that the three largest Czech cities are split into several constituencies (Prague (10), Brno (4), Ostrava (3), see Fig. 1), for which we do not observe pandemic and population data separately, we define 3 new aggregate constituencies, considered all as treated, which cover the full areas of these cities. For two cases in which a constituency is not contained within city limits and includes also other multiple adjacent municipalities, we keep the original constituency numbers assigned to these municipalities. This leaves us with 69 constituencies (69 = 81 − 10 − 4 − 3 + 3 + 2) examined in our analysis.

7 The *t*-test cannot be performed for outcomes observed at the commune level, as communes cannot be exactly classified into voting and non-voting units.

8 We set *n* to 14 days so that the inspected growth rates capture (i) the incubation period, with a median time of 5 days from exposure to symptoms according to Lauer et al. (2020), but which can last up to 12 days, (ii) likely delays associated with seeking testing and acquiring lab results, and (iii) likely delays until a potential hospital admission. For robustness purposes, we define also 7-day growth rates, which similarly as the 14-day growth rates can account for weekly cycles in the pandemic incidence.

9 In our study, we do not estimate the impact of elections on mortality growth rates. It is not feasible in our setting, given that even during the peak of the second pandemic wave in November 2020, the highest count of COVID-related deaths reached around 250 cases per day, i.e. around 0–2 in each of 206 communes. This precludes calculating mortality growth rates, especially in the pre-treatment period.

10 For the three largest cities, the entire city populations are treated as residing in voting constituencies. Our estimates are robust to approximating their population residing in voting constituencies by the share of eligible voters living in voting constituencies.

11 An *F*-test after the event study regression in Eq. (1) for the joint significance of the interaction terms in the third week after elections yields an *F* statistic of 2.64 (*p* = 0.0178).

12 According to the data from the Czech Ministry of Health, approximately 1.6% of all detected cases in Czechia resulted in an eventual death. If we applied a simple direct proportionality, our estimates would thus imply that the elections-related acceleration in the pandemic growth resulted in at least additional 139 COVID-19 deaths, but more realistically in around 172 deaths, if we count also the election impact in part of the three largest cities. This calculation omits deaths undetected by the national tracing and testing system. Figure 9 in the Appendix provides evidence suggestive of the failure of the Czech health system to detect all COVID-19 cases from the second wave of the 2020 pandemic. The figure shows that even after subtracting all official COVID-19 deaths from all-cause mortality in the second half of 2020, there still remains significant unexplained excess mortality in weeks 42–47, exactly during the peak of the second pandemic wave.

13 We use the 2016 regional elections for comparison, because these were the last elections in comparable population. The 2018 Senate elections were held in a different rotation of constituencies, while the turnout in 2018 municipal elections might be dictated by the different nature of the elected office.

14 In Fig. 13 in the Appendix, we provide evidence that the pandemic acceleration is higher in municipalities which had below-median turnout in the earlier regional elections compared to municipalities above the median. We test that the difference is statistically significant at the 1% level 5–10 days after elections. This seemingly counter-intuitive evidence likely reflects that lower voter turnout correlates with various socio-economic characteristics that are key for faster pandemic spread. The evidence suggests that using turnout as an explanatory variable to estimate the impact of elections on pandemic development requires advanced econometric methods that can address the likely bias due to the confounding factors that affect participation in elections and simultaneously drive faster pandemic progress.

## Code availability

All replication files will be made publicly available upon acceptance.
